# Patient and public involvement cultures and the perceived impact in the vulnerable context of palliative care: A qualitative study

**DOI:** 10.1111/hex.13186

**Published:** 2021-01-06

**Authors:** Inge Melchior, Anouk van der Heijden, Esther Stoffers, Frits Suntjens, Albine Moser

**Affiliations:** ^1^ Zorgbelang Limburg (currently Burgerkracht Limburg) Sittard the Netherlands; ^2^ Zuyd University of Applied Sciences Heerlen the Netherlands; ^3^ Maastricht University Maastricht the Netherlands

**Keywords:** cultures, impact, palliative care, patient and public involvement, patient participation, qualitative research

## Abstract

**Background:**

Cultural values are crucial to the practice and impact of patient and public involvement (PPI) in research.

**Objective:**

To understand different PPI cultures among research teams and the impacts of PPI associated with each culture type.

**Design:**

A participatory action research design.

**Setting and participants:**

The setting was 10 palliative care research projects. Seventeen patients and members of the public and 31 researchers participated.

**Intervention:**

A programme consisting of four components: (1) training and coaching of patients and the public to prepare them for participation in research, (2) tailored coaching of the 10 research teams over 12‐18 months, (3) a community of practice, and (4) a qualitative evaluation.

**Results:**

We identified three cultures types: relationship cultures, task cultures, and control cultures. We identified four areas of impact: the project aim became more relevant to the target audience, methodological reliability increased, the research products were better able to reach the public, and the awareness increased, associated with behavioural changes, among researchers regarding PPI.

**Discussion:**

A relationship culture appears to be long‐lasting due to impacting the behaviours of the researchers during future projects. Different cultural types require different types of patients and researcher participants, assigned to different tasks.

**Conclusions:**

Further research remains necessary to investigate the support required by researchers to enable relationship‐ and task‐oriented PPI cultures.

**Patient or public contribution:**

Patient advocates and representatives contributed to our research team throughout the entire research process, as well as within the 10 implementation projects.

## 1 INTRODUCTION

Patient and public involvement (PPI) refers to the involvement of health services users, such as patients, caregivers or community members, in research and is increasingly practiced and studied. Involvement is defined as

an active partnership between the public and researchers in the research process. Active involvement may take the form of consultation, collaboration, or user control. Many people define public involvement in research as ‘doing research ‘with’ or ‘by’ the public, rather than ‘to’, ‘about’ or ‘for’ the public’. This would include, for example, public involvement in advising on a research project, assisting in the design of a project, or in carrying out the research.^1^

The Canadian Institute for Health Research's Strategy for Patient Outcome Research (SPOR) adds that PPI is about *‘meaningful’* collaboration, including governance, priority setting, conducting research, knowledge translation, and evaluation.^2^ Shippee et al distinguished four essential components for meaningful PPI: 1) patients should be involved early on, with a clearly defined role; 2) researchers and patient representatives should be able to develop equal partnerships; 3) patient representatives should co‐learn and be prepared for their tasks; and 4) PPI should be systematically evaluated to improve its impact.^3^

Several systematic studies have provided insights regarding the facilitators and barriers to PPI implementation.^3^, ^4^, ^5^ One factor that is often mentioned is culture. Several reviews, such as Shippee,^3^ Manafo,^4^ and Chambers,^5^ have mentioned relationships, a culture of involvement, and values as being components of PPI culture; however, these reviews have not provided an in‐depth investigation into the impacts of PPI culture.^5^, ^6^ Based on current literature regarding organizational culture, PPI culture can be defined as how power is organized, ranging from concentrated to shared power, and the types of social relationships that are formed between patients and researchers.^7^

Studies focused on PPI impacts have mentioned the relevance and appropriateness of the project to the users, the quality of the research, and improved relationships between researchers and communities.^1^ Manafo et al^4^ distinguished between three impact levels: PPI impacts in the near‐term (the individual level), the intermediate‐term (organizational level) and the long‐term (systemic level). Chambers et al^5^ found that the effects of power, diversity, and emotions, which are all related to our understanding of culture, are especially magnified when PPI is implemented in palliative care research. They concluded that evidence supporting the impacts of involvement was limited; however, when implemented effectively, PPI can benefit all stakeholders by improving the relevance and quality of research. Because PPI impacts have been poorly reported in previous studies,^4^, ^8^, ^9^ we aimed to identify different PPI cultures and to assess the impacts of each culture type in 10 PPI projects in palliative care research.

## 2 METHODS

### 2.1 Design

This qualitative study applied a participatory action research design^10^ to create new knowledge and change practices. We implemented PPI in ten palliative care research projects. This study was performed as participatory research because we did not study the participants from ‘outside’ or ‘above’; instead, we collaborated with patients, the public, and researchers. Participatory action research acknowledges the nature of a research study as a complex social process that yields knowledge within practical contexts.

### 2.2 Context

The goal of our two‐year research project, entitled ‘PPI in 10 palliative care research projects’ was to strengthen sustainable PPI in palliative care, including research, education, and practice (see Table 1).

**TABLE 1:** Description of the participating research projects

Project	Goal	Phases of the project	Involvement of lay people: who and in what?	Involvement of more experienced / professional patient representatives
Project 1	The goal was to improve the palliative care competencies of undergraduate medical students.	Phase 1: reaching consensus on the basic competencies required for medical professionals, using interviews and Delphi rounds. Phase 2: development of a toolbox of educational materials. Phase 3: implementation of the toolbox in the undergraduate medical curricula in the Netherlands. Phase 4: evaluation of the effects of the toolbox.	Interviews with patients in palliative care on the care they receive(d). Two patients not receiving palliative care were involved in sounding board groups during the entire project. They gave input on recruitment, the interview guide, the required competencies, and the educational materials in the toolkit.	Patient advocates were involved in specific activities, such as recruitment, providing educational materials, and disseminating information in their newsletter to reach the wider public.
Project 2	The goal was to improve the quality of advanced care planning in primary care.	Phase 1: exploration of the state‐of‐the‐art by performing a literature review and through expert interviews. Phase 2: development of a guideline for advanced care planning for healthcare professionals (instructions and training). Phase 3: test and evaluation of the guideline. Phase 4: improvement of the guideline. Phase 5: dissemination and implementation of the guideline.	Elderly local people were involved as research participants in the pre‐ and post‐measures of the pilot testing. Family members were involved in preparing the research proposal by participating in a survey to help define the research focus.	Representatives of a regional elderly association played two roles: Representatives participated in the project group and were involved in every phase and step as partners. Representatives gave advice on the pilot testing and the pre‐ and post‐measures and were consulted to validate the results.
Project 3	The goal was to integrate advanced care planning in the care for intellectually disabled people, in personal care plans and communications between clients, family members, and healthcare professionals.	Phase 1: literature study. Phase 2: exploration of current practice. Phase 3: in‐depth interviews with patients, family members, and healthcare professionals about their wishes and expectations. Phase 4: development of an advanced care planning programme (methods and training). Phase 5: pilot and evaluation of the programme. Phase 6: repetition of Phase 2, and comparison of the results. Phase 7: implementation and dissemination.	Patients not in the palliative phase were involved as participants in the interviews in Phase 3. Family members were involved as participants in the interviews in Phase 2 and 3. Three of them were also members of the advisory council and gave feedback on the research process, participated in co‐creation and implementation (Phase 4‐7).	Members of institutional client councils were informed of the progress and (preliminary) results of the project.
Project 4	The goal was to implement pro‐active care planning in elderly care in a regional consortium consisting of 18 cancer networks.	Phase 1: pro‐active care planning was explored. Phase 2: training of healthcare professionals in pro‐active care planning. Phase 3: studying the patient files of patients who were treated by healthcare professionals who followed the training. Phase 4: conducting questionnaires and interviews with patients and caregivers.	Representatives of the general public, with interest in palliative care, were involved in the sounding board group to give input on the research proposal and in the further research process (Phase 0‐4). Family caregivers and patients who had experience with palliative care were consulted in a questionnaire (Phase 4).	A patient advocate (Zorgbelang) was involved in the steering committee: in the preparation of the research proposal, recruitment for the survey, the design of patient involvement, and the execution, by advising the consortium. An experienced patient with interest in palliative care was involved in the pre‐ and post‐analysis of the data, the presentation of the results, and the coaching.
Project 5	The goal was to improve the competencies of newly graduated bachelor's degree and vocationally trained nurses for the provision of palliative care and to ensure that lecturers are skilled in teaching palliative care.	Phase 1: exploration of the current curricula regarding palliative care and development of a competency framework based on interviews. Phase 2: making an overview of available teaching and faculty development material. Phase 3: development and implementation of learning resources.	Patients in the palliative phase and caregivers were interviewed in Phase 1.	Patient representatives (Zorgbelang) were involved in the steering committee during the entire project. They gave feedback on all research decisions, on the developed educational materials and to the lecturers. Patient advocates were involved in the dissemination of educational materials, provided feedback on the competency framework, and provided training materials regarding how to train patients in the teaching role.
Project 6	The goal was to obtain insight into the decision‐making process associated with deactivating an ICD, how often deactivation occurs, and the impacts of non‐deactivation during the process of dying.	Phase 1: exploration of patient files and supplementary files of deceased ICD patients. Phase 2: focus group discussions with patients, family caregivers, and healthcare professionals. Phase 3: a survey among family members of deceased ICD patients regarding the course of dying and their experiences during the last phase of life. Phase 4: recommendations formulated for the national ICD guidelines.	ICD patients not in the palliative phase and their caregivers participated in focus group discussions (Phase 2). Family members of deceased ICD patients participated in the survey (Phase 3).	Members of the patient and family members/caregivers advisory council of the regional consortium on palliative care and the representative of the national ICD patient organization were involved as members of the project group in all phases of the project. Their task was to advise the researchers. The representative of the national ICD patient organization also gave advice on the research design during preparation.
Project 7	The goal was to improve palliative care by developing and implementing the guideline: Palliative care for people with Parkinson's disease	Phase 1: exploration of the palliative care that was provided to patients with Parkinson's disease. Phase 2: interviews with patients and their family members, to gain insights into the experiences of palliative care. Phase 3: writing the guideline. Phase 4: dissemination of the guideline.	Patients with Parkinson, family caregivers and caregivers of deceased patients with Parkinson were interviewed (Phase 2). The general members of the Parkinson's Association were involved by being informed of the results of the project (Phase 4).	Patient representatives of the Parkinson's Association were involved in the steering committee and project team, from drafting the research proposal to dissemination of the results (Phase 1‐4).
Project 8	The goal was to develop a surprise question screening tool for application to a diversity of chronic diseases by medical specialists, family physicians, and geriatricians.	Phase 1: a literature review to identify prognostic factors. Phase 2: exploration of patients’ care experiences when the end was nearing. Phase 3: exploration of healthcare professionals’ experiences on end‐of‐life dialogues with patients. Phase 4: development of a checklist of prognostic factors. Phase 5: implementation of the checklist in three hospitals. Phase 6: statistical analyses on the combination of the most likely prognostic factors and the development of an easy‐to‐use screening tool. Phase 7: development of an e‐learning module and a workshop to support medical doctors in the dialogue regarding nearing the end. Phase 8: implementation of the screening tool, the e‐learning module, and the workshop in three hospitals.	Patients in the palliative phase were involved as participants in the interviews (Phase 2).	A representative of the patient and the caregivers advisory council of the regional consortium on palliative care was involved in validating the research proposal during preparation. Another representative was a member of the project group and was involved in all phases of the project. Another patient representative gave advise on the e‐learning module and the workshop.
Project 9	The goal was to improve the quality of multi‐cultural palliative care across all four dimensions of palliative care and to strengthen the self‐determination of non‐Western migrants.	Phase 1: interviews and focus group discussions on the information needs, norms, values, and wishes of non‐Western migrants. Phase 2: development of educational meetings in collaboration with non‐Western migrants and healthcare professionals. Phase 3: the train‐the‐trainer stage with representatives of non‐Western patient organizations and non‐Western migrant national networks. Phase 4: implementation of educational meetings by trained patient representatives in network meetings of non‐Western organizations and non‐Western migrant national networks. Phase 5: evaluation of the contents and educational meetings.	Non‐Western migrants (citizens, not patients) were involved receiving the educational meetings and during the evaluation (Phase 4 and 5).	Representatives of non‐Western patient organizations and non‐Western migrant national networks played several roles: as equal partners in the project group and as advisees in the advisory committee (advice on the development of the educational meetings, including the information package and workshops, and commented on the final research report). As research participants, they participated in the interviews and focus group discussions (Phase 1). As implementation fellows, they were involved in the co‐design of the educational meetings. They co‐developed and implemented the train‐the‐trainer course. Those who received the train‐the‐trainer course organized and implemented the educational meetings as workshop leaders.
Project 10	The goal was to obtain insights into the current practices of palliative emergency care provided by family physicians and the development of implementation strategies.	Phase 1: exploration of current care by literature, interviews, and focus group discussions. Phase 2: development of a national guideline and implementation plan. Phase 3: implementation of training materials by the Dutch Association of Family Physicians.	Patients receiving palliative care and family caregivers were involved in data collection by participating in interviews.	Patient representatives and patient advocates (Zorgbelang) were involved in the sounding board meeting of the regional consortium on palliative care during the preparation of the research proposal. They were also involved throughout the project, as members of the advisory council. They provided advice on data collection and the development of the guideline.

Note

Patients are people who are in need of palliative care services. (Family) caregivers are those who are or were involved in providing palliative care, such as spouses, children, friends, neighbours or volunteers. Patient representatives are linked to a local patient group or organization. They represent the stakes of specific patient groups or people in need of palliative care and/or have experience in palliative care, such as family caregivers/family members, etc. Patient advocates are staff members of an umbrella organization, operating at the provincial level. Members of institutional client councils are generally family members or residents of a specific healthcare institution. Self‐nominated person advocates are based on personal interest, without being members of a patient organization or institution. Patients with interest in palliative care are chronically ill individuals who require health care, are linked to an umbrella organization operation at the provincial level, and advocate for people in need of palliative care. In the article, we refer to all of these groups as ‘patients’ and distinguish only where necessary.

Our project consisted of four components. Component I centred on the training and coaching of patients and the public to prepare them for participation in research. In collaboration with an experienced patient research partner, we provided three regional training sessions to meet the needs of the patients and the public. Component II focused on the tailored coaching of the 10 research teams, over 12‐18 months, to facilitate the implementation of PPI. In total, we held 35 coaching meetings, which each lasted 1.5 hours, in which we worked interactively through serious gaming, using the ‘participation game’ and ‘participation matrix’ developed previously.^11^ Serious gaming supports the dialogue about PPI and can be used in all phases of a research project. The ‘participation matrix’ was used to visualize PPI and to document the design, planning, evaluation, changes, and contextual influences that both support and hinder PPI during the lifetime of a palliative research project. Component III involved the development of a community of practice. We organized four meetings, at which the involved patients and researchers shared the challenges and opportunities they encountered during their projects. Component IV was the qualitative evaluation of the perceived impacts of PPI implementation.

### 2.3 Participants

The participants in the 10 projects included researchers (sometimes referred to as healthcare/educational professionals) and patients, caregivers, patient representatives, and patient advocates, which will collectively be referred to as ‘patients’ for the sake of readability. The only inclusion criterion applied in our study was involvement in one of the 10 projects. A total of 17 patients participated in this study, three of whom were male and 14 of whom were female, aged 29‐82 years. The patients’ experiences with PPI varied from first‐time involvement to several years of PPI. Of the 31 researchers, 27 were female, 10 were junior researchers (less than 2 years research experience), and 21 were senior researchers. Their research experience ranged from 0 to more than 20 years. Nine projects were performed at five universities throughout the Netherlands, and one was performed at a knowledge institute with close university ties.

We used two sampling strategies: self‐selection for projects and purposive sampling for patients involved in the projects. The senior researchers associated with each project recruited patients for this study.

### 2.4 Ethics

This project was reviewed by the ethical review board at Zuyderland ‐ Zuyd University (16‐N‐108). Participants signed informed consent forms. Participants received verbal and written information describing the goals of the study and how confidentiality and anonymity were assured.

### 2.5 Data collection

Data were collected between September 2016 and June 2018 using a multi‐method process, including observations, field notes, informal conversations, and in‐depth interviews. We made observations during all 35 coaching workshops (AM, IM, AvdH, ES). In addition, the coaching meetings were audiotaped and transcribed verbatim. Fieldnotes (AM, IM, AvdH, ES) were taken during the participation community of practice meetings, which focused on the design of the PPI, contextual factors, and solutions to practicalities. We collected data during informal conversations (AM, IM, AvdH, ES) throughout the project, in the form of emails, memos from phone consultations, and informal talks. At the end of the project period, we conducted 20 in‐depth interviews, 10 with patients, and 10 with researchers. We asked the interviewees about the perceived impacts of PPI, and the barriers, facilitators, and strategies that strengthened PPI. We used the ‘most significant change’ interview method (IM, AvdH).^12^ We used an open approach, allowing the interviewee the opportunity to decide their stories.^13^ The interviews lasted from 30‐90 minutes, and all interviews were audiotaped and transcribed verbatim.

### 2.6 Data analysis

In the analysis, we applied the model of organizational culture.^7^ Machado described a model with two axes: one axis ranging from shared to concentrated power and the second axis ranging from personal to functional relationships within the organization. Machado^7^ distinguished four organizational cultures: (a) the family business (personal relationships and concentrated power), (b) bureaucratic cultures (functional relationships and concentrated power), (c) result‐driven cultures (functional relationships and shared power), and (d) personal satisfaction cultures (personal relationships and shared power). First, we analysed the data for each project team to determine how power was exercised. Power was characterized as either concentrated or shared, depending on the extent to which equality was valued during the project and whether decisions were made collectively. Second, we analysed the data regarding the nature of human relations. We divided the projects into cultures that were driven by subjective, emotional, and personal values (personal) and cultures that were driven by objective, rational, and functional values (functional). Using constant comparison,^14^ the 10 projects were categorized according to their PPI cultures. We also examined the perceived impacts for each project, as defined by the patients and researchers during the interviews, to explore how culture and perceived impact relate to each other.

### 2.7 Trustworthiness

Member checks were used to increase the credibility of the study. We sent the participants summaries of our analyses for feedback. We also used data triangulation (the literature, the experiences of patient representatives, advocates, and researchers), methodological triangulation (observations and interviews), researcher triangulation (analyses by different researchers), and peer debriefing (discussing the applied codes). To guarantee the external validity, we used thick description. We described the context of the project, the project characteristics, and the selection of participants.^15^

### 2.8 Patient and public involvement

Our project group consisted of three researchers (two junior and one senior), one patient advocate (a staff member of Zorgbelang Limburg, which strengthens patient and public involvement in the province of Limburg, the Netherlands), and two patient representatives, who were also affiliated with Zorgbelang Limburg. One was the chair and founding member of the regional Patient Sounding Board Palliative Care and has extensive experience in patient representation. The other has been involved for several years with a volunteer‐managed hospice. He had no previous experience in PPI in research but brought experience as a member of various committees from his professional past. The project was based on co‐leadership and co‐responsibility, and PPI was implemented at every phase.

## 3 FINDINGS

### 3.1 Participation cultures

We identified three culture types: relationship cultures, task cultures, and control cultures. We will first discuss the most participative PPI culture and finish with the most traditional PPI culture. We use pseudonyms to ensure the anonymity of participants.

#### 3.1.1 Relationship cultures

We defined a relationship culture as being based on personal relationships between patients and researchers, with genuine power sharing. The patient representatives in this PPI culture are emotionally close to the patients requiring palliative care—they are either themselves patients (who are not receiving palliative care), elderly or caregivers—and are generally patient representatives by experience rather than by profession. Projects 2, 5, and 7 are examples of this culture type, in which the researchers found investing in a personal relationship with the involved patients to be important. The researchers were interested in the patients’ lives and aspirations and were open to adapting to the patients’ lives.

Guy, a researcher for Project 5, argued that researchers are often reluctant to engage in research with patients because they find it daunting, or they assume that patients are too ill to be involved. Therefore, he stressed that establishing a personal relationship with involved patients is important:

‘I see that researchers find it hard to ask patients for a favor, as they participate voluntarily, and we are afraid to ask too much from them. Discovering that patients do not see it as doing us a favor, but they want to participate, was really an eye‐opener for us. If you have a personal relationship with people, it is easier to ask someone, and they will feel freer to say no’.

In general, Guy did not appear afraid to listen closely to patients and respond flexibly. He was not afraid of lacking full control when confronted with ‘the other’. The ability to truly listen to patients and adapt accordingly is a crucial aspect of this culture type.

‘These interviews really impressed me, I felt emotionally touched. While conducting the interview, I immediately decided to throw overboard the interview guide, which we, as researchers, had constructed beforehand. Checking questions does not fit when you talk to someone in such an important phase of his/her life. I decided to just listen. […]’

Hanna, a researcher for Project 7, acknowledged that a traditional project culture, in which the professional makes the decisions, does not always work and that a flexible approach is necessary.

‘For instance, it is difficult for Dorry [patient representative] to be physically present at meetings because her husband cannot stay alone at home. Then we try to schedule a phone meeting’.

This provided Dorry with the feeling that she really belonged to the project and that she was needed, and they could not do without her. In this type of participation culture, team spirit and coherence are strong. All participants believe that they must work together to accomplish their goals. The patients are not only consulted or asked to give advice; they participate as partners in the project or the steering group. The power is shared in these PPI cultures. Willy, the representative of an elderly organization involved in Project 2, provided this summarization:

‘I felt really welcome. And useful. I did not have the feeling that I participate because ZonMW [the funding organization] requires that, but because the researchers sincerely want to learn from us patient representatives. […] I have been engaged as a full member. They listened when I had ideas. And we made most decisions collectively’.

#### 3.1.2 Task cultures

We defined task cultures as functional relationships between researchers and patients, with task‐oriented team positions and selectively delegated power. Projects 8 and 9 showed these cultural traits. In contrast to relationship culture, the patient representatives and researchers have functional relationships: the patient representatives are selected for their professional experience as patient advocates—in their professions, they are close to the public—to fulfil a clear task within the project team.

Jelena, a researcher for Project 9, presented a very clear division of labour for their project. They engaged with several semi‐professional and professional migrant and patient representatives during various research phases to ensure that the developed educational material would fit the target audience. They also consulted citizens with migrant backgrounds. The patient representatives were allowed complete freedom within the tasks assigned to them. Halima, who had a migrant background and was a professional educator for migrant groups, was involved as a patient representative, tasked with educating citizens with migrant backgrounds on palliative care. She was trained by the researchers to provide palliative care education. Although Jelena would be present during the meetings with patients, she did not interfere with Halima. Halima felt taken seriously because she had freedom within the task assigned to her:

‘Educating this target audience is my job. Only the topic of palliative care was new to me. This is a difficult topic to discuss in many migrant cultures. Due to my professional experience with these groups, I knew, for instance, that I needed to focus especially on the family circumstances of the patients, more than on the patients themselves, as the collective is very important for this target audience’.

This result was the goal Jelena tried to achieve by giving Halima ‘her space’ within the project:

‘If someone shines, one's motivation will be biggest and one's participation most impactful’

During Project 8, the task division between the researchers and Lisa, the patient representative, was also clear. Lisa was asked to provide a critical eye to review the work performed by the researchers. She did not receive pre‐defined tasks but experienced the freedom to take a pro‐active role. For instance, she presented the idea of performing a pilot interview and arranged an interviewee for this purpose. Her ideas were taken seriously by the researchers, who gave her the opportunity to contribute freely.

In contrast to the relationship culture type, the contributions that the researchers expected from Halima and Lisa were not to provide a lay or ‘authentic’ perspective, based on the actual experiences of people requiring palliative care but, instead, to provide their critical perspectives, based on their professional experience as advocates. Halima was a professional trainer of migrant groups, whereas Lisa was a former social worker and a member of the regional patient and caregivers advising council.

#### 3.1.3 Control cultures

We defined control cultures as based on control exercised by researchers, who acted as the traditional powerholders and the responsible individuals for the project. They invited patients to participate in roles defined by the researchers.

The third PPI culture type, which is also the most traditional type, characterized Projects 1, 3, 4, 6, and 10. In these projects, the patients and researchers have functional relationships similar to those in task cultures. However, in control cultures, power and control are not shared but remain with the researchers. The involved patients are experienced experts, caregivers, professional patient advocates, and members of client councils. The patient representatives were not selected for their unique individual experiences or professionalism but to provide general feedback from a lay perspective in response to the ideas of the researchers. They primarily participate on the advice level.

The patients do not refer to ‘our project’. By providing feedback, they feel they are doing the researchers a favor. In addition, the researchers view the project as being ‘theirs’. During Project 10, researcher Pat considered the patients to be potentially useful for confirming their decisions and as a special *addition* to the project but does not view them as an *essential* project component.

‘I liked the fact that the target audience [of our project] confirmed what we [researchers/professionals] already knew. […] The advisory board gave suggestions to adapt the survey. We also asked them for advice on the final report, for which they made small suggestions. They confirmed we were on the right track’. (researcher, Project 10)

The power does not shift, and relationships in control cultures have a traditional form. During Project 3, Jet, who has been an active participant on health organizational boards since she had a baby with Down syndrome 34 years ago, was a very outspoken personality, who made an attempt to actively provide input for the project. She suggested ideas for recruiting more patient representatives and obtaining more patient input, a problem that the researchers were struggling with because they did not have the necessary network. Jet was willing to use her own social network. However, the researchers did not accept Jet's ideas or inform her of their reasoning. This left Jet feeling that they were not really waiting for her input. She felt that her involvement was a form of lip service.

Patients in this culture type participate in meetings and respond to the questions asked by the researchers but generally do not feel invited to pro‐actively engage with the project. The researchers in these projects are often novices to PPI and choose to use advisory boards as the most established method of patient involvement to preserve their power.

### 3.2 Participation cultures and PPI impacts

To subsequently determine the PPI impacts of the three culture types, we examined what the patient representatives were able to accomplish in the various projects and how they and the researchers perceived their impacts to be meaningful. Based on our data, we discerned four areas of impact: 1) the project aim became more relevant to the target audience, 2) methodological reliability increased, 3) the research products were better able to reach the public, and 4) awareness increased, associated with behavioural changes, among researchers regarding PPI.

#### 3.2.1 The project aim became more relevant to the target audience

In all three PPI cultures, this impact was evident. In relationship cultures, the project was perceived to be more relevant because the involved patients know from experience what the target audience views as important, and they have a social network that can be consulted. For instance, Dorry, a caregiver and a member of the patient organization (Project 7), felt that she could really have an impact on the relevance of the project:

‘I shared my experiences as a caregiver […], and I functioned as a bridge with the [patient] organization. I gave an interview about the project in the members’ magazine. And I knew that the [patient] organization was making a film about the palliative phase, and they had the same idea within the project. So, I introduced them to each other’.

In task cultures, the patient representatives also impacted the project relevance because they were professionally close to people who required palliative care and were able to advocate for their stakes. In control cultures, the patient representatives might not have a pro‐active say in the project aim; however, the researchers do need to be able to legitimize their project to the patient representatives. Therefore, the researchers remain obligated to consider the patient perspective, at least to a certain extent.

#### 3.2.2 Increased methodological reliability

In all three PPI cultures, the projects have become methodologically more reliable because the involved patients provided a lay perspective on the materials used, such as recruitment letter and survey questions. They speak the same language as the intended audience. Guy, a researcher working on Project 5, added that a personal relationship between the patients and the researchers was essential to allow the researchers to feel comfortable inviting the patients to discuss methodological issues.

Halima, working on Project 9, previously trained many patients with migrant backgrounds. She mentioned that she warned the researchers to avoid including discussion topics that are considered ‘taboo’ in certain cultures. During Project 2, Willy proposed to perform a pre‐test for the questionnaire and arranged test‐persons for this purpose, which improved reliability. Dorry (Project 7) was able to recruit many people due to her social network with the patient organization. Patients were more willing to participate when someone ‘from inside’ asked them to do so. Hanna, a researcher working on Project 7, noticed that interviewee recruitment was much easier when working with patient representatives who have close ties to the target audience, resulting in a more diverse sample.

In control cultures, the researchers mentioned an impact on the methodological reliability, although the patients themselves often state they are unsure whether they have made any impact because they are not informed by the researchers how their feedback is used. Sometimes, the researchers in this project culture can neglect opportunities. For instance, Jet, the involved caregiver in Project 3, recalled an instance where the researchers showed her film recordings of interviews that had been performed with clients presenting mental disorders, regarding the ends of their lives.

‘The clients were interviewed by their own healthcare professionals. On the one hand, that is, of course, a relationship of trust. On the other hand, that is also an inherently unequal relationship. The language used was also too complicated, and questions were suggestive. But they showed the film only after they had conducted the interviews. I was not consulted before, so I could not have any impact’.

#### 3.2.3 Research products were better able to reach the public

In the relationship and task cultures, the participants also perceived that the final products were better able to reach the public. Patients feeling invited to take pro‐active roles were key to this impact.

During Project 5, Maria, the patient representative, was involved in the implementation phase and wrote the press release. Dorry (Project 7) published the project results in the magazine of the patient organization, and co‐wrote and co‐disseminated the guidelines to relevant partners, such as health professionals and professional bodies. Because the projectgroep made the film on palliative care in close cooperation with the patient organization, reaching the public was very easy.

During Project 9, Halima used her professional network to reach the public. She previously worked with various ethnic minority groups. Therefore, once the researchers finished making a film for Chinese people and another film for Antilleans and Arubans, Halima had the necessary contacts to deliver these films to these populations.

Project 1 is an interesting example because it slowly changed from being a control culture to being a task culture. At first, Jan, the patient, was given a seat on the advisory board. After one year, the researchers concluded that this was not where the patients’ contributions should be.

‘It is difficult to not lose the patients’ voice in meetings with policymakers, managers, and educational experts’.

Frieda, the researcher working on Project 1, discovered that Jan wrote columns for the website of a local cancer organization. Frieda asked Jan whether he would like to do something similar for their project website. Jan perceived this as an opportunity to actually have an impact:

‘I like to write. I feel I can have an impact on how other cancer patients experience their disease. Maybe I can provide them some support to let them know they are not alone’.

While on the advisory board, Jan had no idea of what the researchers did with his advice. Since Frieda has asked him to write the columns, he has felt more integral to the project and is now also pro‐actively contributing:

‘I have been a drama teacher and film director, and for most of my life, I have been teaching. I would like to contribute to the project with that experience. I could make films and educational material with patients in the palliative phase, which can be used in the education of medicine students’.

#### 3.2.4 Increased awareness, associated with behavioural changes, among researchers regarding PPI

The relationship culture was the only culture type to increase the awareness of the researchers regarding the value of PPI. Researchers positioned themselves as being equally vulnerable as the patients. Guy (Project 5) recalled how an interview in hospice changed his general way of thinking and working:

‘I decided that they [the patients] are so essential that if they cannot make it to a meeting, we have to cancel the meeting. There is no point in meeting with only professionals. We no longer do that’.

His colleagues were initially surprised when he cancelled a project meeting due to the lack of attendance by patient representatives. He decided that researchers should no longer talk *about* people without involving them. This awareness grew wider than just PPI. Guy, also being a lecturer, decided that lecturers and teachers also no longer should decide on educational matters without involving students.

The researchers working on Projects 2 and 7 also mentioned how ‘normal’ involving the public has become for them after experiencing the value of PPI during this project. Therefore, in relationship cultures, PPI might have a more sustainable impact on the future projects of these researchers.

## 4 DISCUSSION AND CONCLUSION

The goal of the current study was to understand the different PPI cultures and the impacts associated with each culture type. We identified three cultures types: relationship cultures, task cultures, and control cultures. We identified four areas of impact: the project aim became more relevant to the target audience, methodological reliability increased, research products became better able to reach the public, and awareness improved, associated with behavioural changes, among researchers regarding PPI. Projects with relationship cultures appeared to demonstrate impacts in more areas than other cultures, and their impacts appeared to be long‐lasting because they impacted the behaviours of the researchers during future projects. PPI in control cultures, in which patients were only invited to provide feedback in response to the ideas of the researchers, might have some impacts by forcing the researchers to face and explain their projects to the involved patients; however, generally, the involvement in control cultures is often tokenistic, as we found out. Different culture types require different types of patients and researchers, who must be assigned different tasks: participants either contribute with their personal experiences (relationship cultures) or their professional expertise (task cultures) on a partnership level, or they contribute lay perspectives on the level of consultation or advice (control cultures).

Although it may appear that each PPI project has a certain culture, which can be determined and associated with the perceived impacts for that project, the reality is that PPI culture is fluid and complex. Cultures are not static but are dynamic and diverse.^16^ Several projects presented a mixture of the three culture types, involving patients at different levels. In these cases, we classified them according to their most dominant culture. For instance, for Project 2 a group of elderly people were consulted, in addition to Willy. The relationships between the researchers and these other patient representatives were characterized by a control culture. However, we classified this project as having a relationship culture based on the relationship between Willy and the researchers, which was the most dominant participation culture. Other projects, such as Project 1, transformed from a control culture to a task culture, triggered by the coaching we provided. Our coaching meetings forced the project teams to reflect on who would be involved during each project phase and how they involved these individuals.^11^ By engaging in this dialogue, they discovered each other's ideas and strengths.

To a large extent, the impact areas overlapped with those defined by Staley et al,^1^ Manafo et al,^4^ and Chambers.^5^ As expected, the PPI impacts depended on the culture type, and each culture type required different types of patients and researchers, with various positions assigned to them. In both the relationship and task cultures, the researchers and patients worked as partners on the project and steering groups. This relationship could be linked to the partnership described by Arnstein,^17^ which enabled them to negotiate and engage in trade‐offs. In these cultures, the patients’ impacts were perceived to be high because the patients, either by experience (often in relationship cultures) or professionally (often in task cultures), are closely associated with people who require palliative care. They had or represented the lay perspective, and the researchers included the patients’ input. Relationship and task cultures differed in the extent to which long‐term PPI impacts were perceived. Relationship cultures have the potential to make researchers change their belief in the value of PPI and make them implement PPI in future projects accordingly. In task cultures, the more professional patient advocates can be involved as individuals in future projects, whereas this is often not possible with the type of patients (by experience) typically involved in relationship cultures. Thus, our findings show that both relationship and task cultures can make long‐term involvement possible in different ways, extending beyond a single project, which is essential for impact, according to Staley.^1^

In control cultures, patients are primarily involved in advisory boards and often do not have any impact beyond giving advice and legitimizing the work of the researchers. We found several causes to the existence of control cultures. First, some researchers found it hard to open up to the idea that patients might ‘know’ something that they as researchers do not know, which let the researchers to assign them only the role of advising. Arnstein^17^ calls this ‘tokenism’, which allows the patients to be heard but assures the researchers the right to decide. Second, in some projects the researchers involved the ‘wrong’ type of patients at the ‘wrong’ stage of the research, for instance a lay patient with a background in education that is involved in setting up research together with experienced researchers, rather than in the phase of developing educational materials. In those cases, the patients could not reach their full potential and a mind shift among the researchers did not occur. Both causes led to a downwards spiral, because the researchers did not reap the rewards of PPI and they felt confirmed in their assumption that patients cannot be given more agency than in an advising role. Our coaching aimed at breaking the spell of this downwards spiral, but we experienced some barriers in doing so. It was not easy to get researchers out of their comfort zone, in which the idea that ‘research is for researchers’ was dominant. This was especially hard when the researchers perceived us as outsiders interfering into their projects. Consequently, our coaching was especially in control cultures more challenging and in some projects we managed to ‘reach’ the researchers better than in others. We faced the challenge of appreciating their authority while simultaneously gently introducing them to the concept of PPI. We sometimes faced ‘lip service’ and ‘window‐dressing’, but also depended on these project leaders as gatekeepers to organize interviews. Especially in the case of control cultures did our coaching meetings provide unique insights, as we could observe there sensitivities, tensions, and personal issues within the project teams, which were not mentioned during the interviews.

Our findings on PPI cultures and impacts appear to be generally transferable to PPI projects in contexts other than palliative care, especially those associated with people in other vulnerable circumstances (eg with multiple health needs, communication vulnerable people). However, the practice of PPI discussed in this study is specific to the palliative context. Patients who require palliative care are rarely involved in a project group or steering group because they cannot make such long‐term commitments. In these 10 projects, the palliative care patients were involved at the consultation level by being interviewed, which was not associated with either a relationship or task culture. Six of 10 projects (Projects 1, 4, 5, 6, 8, and 10) involved patients in the palliative phase. In Projects 1, 4, 5, and 8, the patients in the palliative phase were interviewed or asked to fill out a questionnaire. In Projects 6 and 10, the (lay) caregivers for palliative care patients were involved, who did not have emotional distance from the palliative care experience. A relationship culture in palliative care appears more likely when caregivers and loved ones are involved, instead of actual patients. Those close to the actual experience who are not constrained by the need for palliative care must be involved, which makes impactful PPI easier to realize for the researchers, who often struggle to ask patients for involvement during this very special and important phase of life.^5^ Arguably, the gap between researchers and patients is larger during the palliative phase than during other healthcare stages, which increases the potential impacts that PPI might have in the palliative context.

These findings provide us with a better understanding of the relationship between culture and PPI impact. Although Shippee et al,^3^ Manafo et al,^4^ Chambers et al,^5^ and Evans et al^6^ identified ‘relationships’, ‘culture of involvement’, ‘values’, and ‘the principal investigator's leadership’ as essential components of PPI, no previous studies explored how culture affected PPI impacts. It even seems that working with PPI created for some researchers a wider culture shift, beyond their PPI project. The story of Guy, who now also involves students in education, shows the wider impact of PPI. Also Frieda mentions she now aims to involve students and patients more to think together about better education, and Ankie (researcher Project 4) mentioned that researchers have learned that the message they convey might not be the same as the message received. It taught her to actively try to put herself in the shoes of ‘the Other’, in her life more broadly.

However, due to scope and time constraints in the current project, we were not able to actually study the wider changes of researchers’ behaviour, including beliefs and values about PPI, in their other research works. We think that the wider cultural changes to improve PPI in research require much more investigation, especially at the national level. In the case of the Netherlands, all research projects funded by the research programme ‘Palliantie’ are obliged to implement PPI. However, the wider cultural change has not been investigated yet. Further research also remains necessary to investigate whether PPI culture is positively or negatively influenced by patients and researchers with more PPI experience. Furthermore, PPI in palliative research has a relatively young history. According to Chambers,^5^ the barriers to impactful PPI are primarily associated with the conduct of the researchers. A deeper exploration of the support required by researchers to enable the development of relationship‐ and task‐oriented PPI cultures could further develop coaching in PPI in order to move further away from control cultures. Including behavioural change elements based on psychological behaviour theories, such as the Theory of Planned Behavior^18^ or more implementation oriented like the Behavior Change Wheel^19^ into PPI coaching, might extend the impact of PPI in both time and space.

## CONFLICT OF INTEREST

No conflicts of interest.

## AUTHORS’ CONTRIBUTION

Inge Melchior has substantially contributed to the data collection, analysis and interpretation, and to the conception, design and writing of this manuscript. Anouk van der Heijden has substantially contributed to the data collection, analysis and interpretation, and to critically revising this manuscript. Esther Stoffers has substantially contributed to the conception and design of this project, its data collection, analysis and interpretation, and to critically revising this manuscript. Frits Suntjens has substantially contributed to the coaching of the 10 projects (our intervention), the data analysis and interpretation during our coaching period, and to critically revising this manuscript. Albine Moser has substantially contributed to the conception and design of this project, its data collection, analysis and interpretation, and to the conception, design and revising of this manuscript.

## Funding Information

This work was supported by the Netherlands Organization for Health Research and Development, The Hague (project number 844001212), as part of the programme Palliantie. Meer dan zorg (Palliantie. More than care).

## Data Availability

The data that support the findings of this study are available on request from the corresponding author. The data are not publicly available due to privacy or ethical restrictions.
